# A transient mitochondrial switch underlies long lasting effects of adolescent cannabis

**DOI:** 10.1038/s41386-026-02539-3

**Published:** 2026-08-27

**Authors:** Christina Ioannidou, Irene Manzanares-Sierra, Daniel Jimenez-Blasco, Darío García-Rodriguez, Israel Manjarrés-Raza, Francisca Julio-Kalajzić, Antonio C. Pagano Zottola, Martina Di Bartolomeo, Guillaume Ferreira, Juan P. Bolaños, Giovanni Marsicano, Arnau Busquets-Garcia

**Affiliations:** 1 University of Bordeaux and INSERM, U1215 NeuroCentre Magendie, Bordeaux, France; 2 Cell-Type Mechanisms in Normal and Pathological Behavior Research Group, Neuroscience Research Program, Hospital del Mar Research Institute, Barcelona, Spain; 3 Institute of Functional Biology and Genomics (IBFG), Universidad de Salamanca, CSIC, Salamanca, Spain; 4 Institute of Biomedical Research of Salamanca (IBSAL), Salamanca, Spain; 5 Centro de Investigación Biomédica en Red de Fragilidad y Envejecimiento Saludable (CIBERFES), Madrid, Spain; 6 U1312 Bordeaux Institute of Oncology, INSERM, Pessac, France; 7 Department of Bioscience and Technology for Food, Agriculture and Environment, University of Teramo, Teramo, Italy; 8 University of Bordeaux, INRAE, Bordeaux INP, NutriNeuro, Bordeaux, France

## Abstract

Adolescent cannabis use produces lasting and sex-specific effects into adulthood, although poorly explained mechanistically. This study links these cannabis-induced effects to sex-dependent changes in phosphorylation of mitochondrial Complex I subunit NDUFS4 during adolescence, identifying mitochondrial pathways as causal mechanisms and potential therapeutic targets to reduce their long-term consequences.

## Introduction

Cannabis is one of the most widely used drugs of abuse among adolescents, a developmental period marked by profound neuro-biological remodeling and vulnerability [1]. Epidemiological evidence has linked adolescent cannabis use to a heightened risk of psychopathology in adulthood, particularly schizophrenia-spectrum conditions involving social withdrawal and working memory deficits [1, 2]. However, the causal mechanistic underpinnings of these long-term effects remain unexplained. Preclinical studies in rodents have recapitulated key features of these associations, showing that, *via* activation of type-1 cannabinoid receptors (CB1) [3], adolescent exposure to the main psychoactive component of the *Cannabis sativa* plant, Δ^9^-tetrahydrocannabinol (THC), can induce persistent alterations in behavior, such as hyperlocomotion, decreased social interaction and working memory deficits. Notably, these outcomes often differ between male and female subjects [3]. However, despite the availability of several animal models, causal studies are still lacking in this field, likely due to the difficulty of following molecular mechanisms over several weeks.

Beyond their canonical location at cellular plasma membranes, CB1 receptors are also functionally present at mitochondrial membranes (mtCB1) in both astrocytes and neurons, where they mediate some of the brain and behavioral effects [4] of THC. In adults, acute THC administration activates mtCB1 in astrocytes, disrupting mitochondrial complex I (CI) activity through decreased phosphorylation of its NDUFS4 subunit, thereby impairing bioenergetic function [5]. Because mitochondrial alterations during development can cause long-lasting brain dysfunction [6], we hypothesized that such mechanisms might be causal determinants of the adult behavioral effects of adolescent THC exposure.

## Material and Methods

### Mice

All experiments complied with the French (Décret 2013-118) and European (2010/63/EU) rules on animal experimentation and were approved by the Committee on Animal Health and Care of INSERM, the French Ministry of Agriculture and Forestry (authorization number 3306369) and the French Ministry of Higher Education, Research and Innovation (authorization APAFIS#15502). Adolescent female and male C57BL/6 N mice were used in these experiments. Sample sizes were chosen based on similar prior experiments. Animals were collectively housed under standard conditions of temperature and humidity in a 12 h light/12 h dark.

### Drugs

THC was purchased from THC-Pharm (Frankfurt, Germany) and dissolved in 4% ethanol, 4% Cremophor-EL and 92% saline. THC (1 or 5 mg/kg) or vehicle were administered once daily by intraperitoneal injection in a volume of 10 ml/kg, from postnatal day (PND) 35 to PND 45.

### Behavioural testing

Behavioral experiments were conducted at PND70.

#### Open-field test

Mice were placed in an open field Plexiglas box (width, 60 cm; length, 60 cm) the bottom of which was divided into squares. Total locomotion was assessed by the number of total squares crossed and the number of vertical rearings.

#### Two-chamber social interaction test

Mice were tested in an open field arena with two containers, one with a mouse (adult C57BL/ 6 N), while the other was empty. ‘Social’ and ‘non-social’ zones were an 8 cm area surrounding the containers. A social interaction index was calculated as time spent in the social zone divided by the total time spent in both zones.

#### Spontaneous alternation

Mice were placed in a Y-maze (42 cm long, 8 cm wide, 120° between arms) in a room with 50 lux of light.

Mice were allowed to explore freely the maze for 8 min. The percentage of alternation was calculated as: [number of correct triplets / (total number of arm entries − 2)] × 100.

### Western blotting

Brain tissues were collected at PND46 and PND70, lysed in RIPA buffer with protease and phosphatase inhibitors, boiled, and centrifuged. Protein extracts (50 μg) were separated by SDS–PAGE and transferred to nitrocellulose membranes. After blocking, membranes were incubated overnight at 4 °C with primary antibodies [mouse monoclonal anti-ATPB (1:1000, MS503, MitoSciences), rabbit polyclonal anti-phospho-Ser173 NDUFS4 (1:100, provided by D. De Rasmo), and mouse monoclonal NDUFS4 (1:800, ab87399, Abcam.)], followed by HRP-conjugated secondary antibodies [horseradish peroxidase-conjugated goat anti-rabbit IgG or goat anti-mouse IgG (1:10,000, Santa Cruz Biotechnologies)]. Signals were detected using chemiluminescence and visualized on X-ray film. Experiments were performed with independent biological replicates, and a representative blot is shown.

### Intravenous viral vector approach

PND25 mice were anesthetized with isoflurane and injected retroorbitally with AAV vectors (5 × 10^10^ viral genomes in 50 μL). To confirm expression, mice were perfused with PBS and formalin, and brains were cryoprotected, sectioned (30 μm), and stored. Sections were incubated overnight with anti-GFAP primary antibody (1:500; DAKO), followed by fluorescent secondary antibody (donkey anti-rabbit Alexa Fluor 647, Invitrogen), then mounted and imaged using an epifluorescence microscope (Leica, Germany).

### Statistical analysis

Data were analyzed with Prism 9.0 (Graphpad Software). Data were checked for normality using the Shapiro–Wilk test. Repeated-measures or unpaired statistical analyses were performed using Student’s t-test or one- or two-way ANOVA, as appropriate, to compare two or multiple groups. For ANOVA, only when significant interaction effects of main factors were detected, post-hoc analyses (Dunnett’s or Sidak’s) were performed. When normality could not be calculated for all groups, non-parametric tests (Mann-Whitney test) were also used. Significance was set at *p* ≤ 0.05.

## Results

**Fig. 1:**
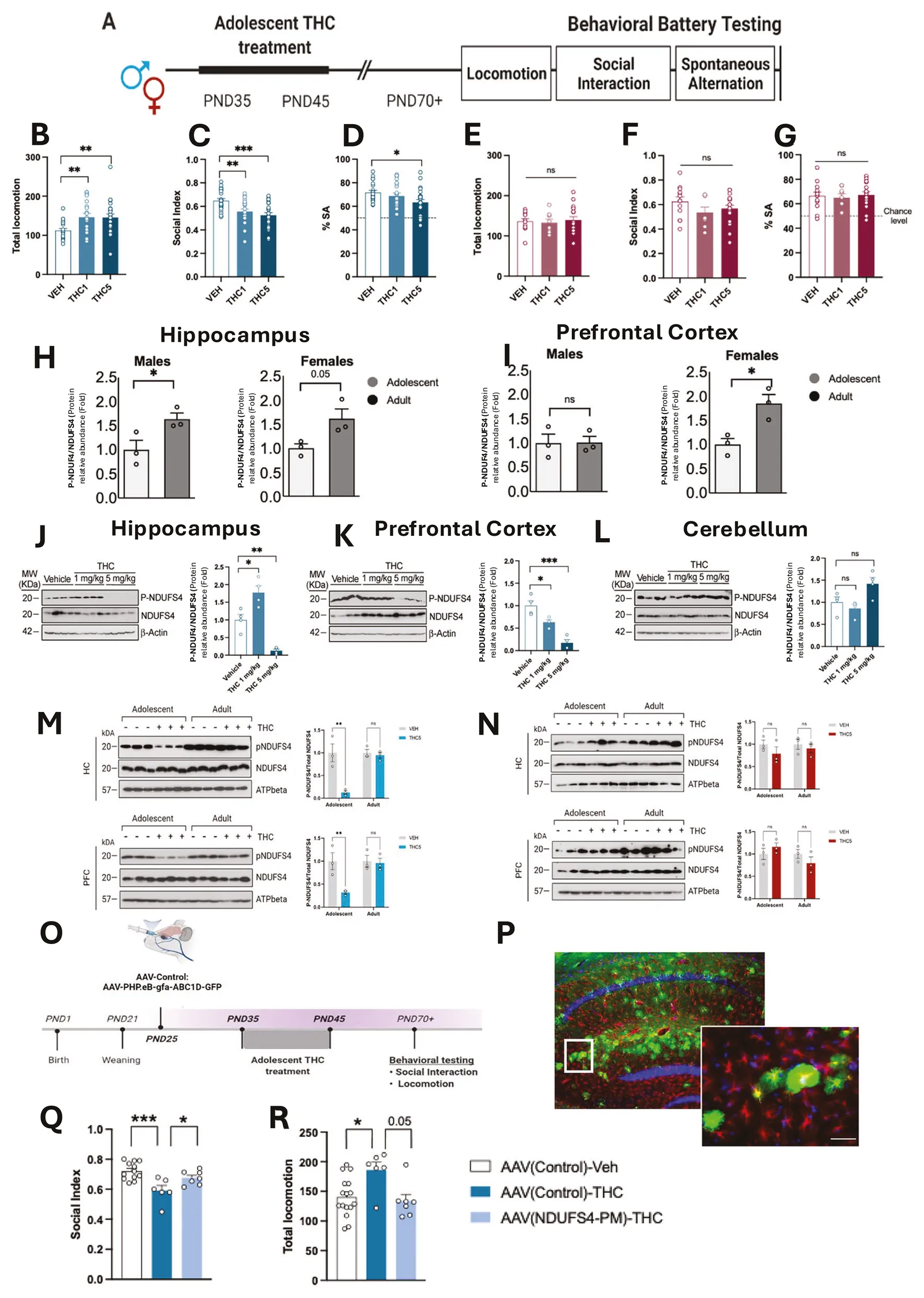
NDUFS4-PM expression in astrocytes blocks the adolescent THC-induced alterations in social interaction and locomotion in adult male mice. **A** Schematic timeline of the experimental design. B–G Effect of adolescent THC (1 and 5 mg/kg) or vehicle in the total locomotion (number of crossings and rearings), in the social interaction task and in the spontaneous alternation (SA) test in male (B–D) or female (E–G) mice. H, I Comparison of phosphorylated NDUFS4 (pNDUFS4) levels in adolescent and adult male mice in hippocampus (H) and prefrontal cortex (I) from the data obtained from gels M and N. J–L Effect of adolescent THC (1 and 5 mg/kg) on pNDUFS4 corrected by total NDUFS4 in the hippocampus (J), prefrontal cortex (K) and cerebellum (L) of male mice. M, N Effect of adolescent THC (5 mg/kg) on pNDUFS4 corrected by total NDUFS4 in the hippocampus (HC) and prefrontal cortex (PFC) in adolescent and adult male (in blue, M) and female (in red, N) mice. The effect of THC is normalized to the respective vehicle group for each condition. O Schematic timeline of experimental design of viral expression of GFAP-NDUFS4-PM by retro-orbital. P Representative images showing expression of AAV-GFAP-GFP in the hippocampus of adult male mice. GFAP (red), GFP (green), and DAPI (blue) in the sagittal section. Scale bar, 200 μm and 40 μm. Q, R AAV-GFAP-NDUFS4-PM blocked the induced decrease of social interaction (Q) and hyper-locomotor activity (R) in adult male mice. All data are presented as mean ± S.E.M. * *P* < 0.05; ** *P* < 0.01; *** *P* < 0.001 compared to mice exposed to vehicle treatment. * *P* < 0.05 (as compared to AAV-Control-Veh (includes AAV-Control-Veh and AAV-NDUFS4-PM-Veh), #*P* < 0.05 (as compared to vehicle treated mice).

We investigated behavioral outcomes in adult C57BL/6 N mice repeatedly exposed to THC during adolescence. Male and female mice received daily intraperitoneal injections of THC (1 or 5 mg/kg) between postnatal days (PND) 35 and 45, a period of heightened neurodevelopmental vulnerability [7]. At PND70 (young adulthood), we assessed locomotion, social interaction and working memory (Fig. 1A). Adolescent THC exposure produced robust alterations in male mice (Fig. 1B–D), including hyperlocomotion, reduced sociability and impaired spontaneous alternation. Interestingly, female mice remained largely unaffected (Fig. 1E-G). Beyond locomotion and cognition, social deficits, which are considered core “negative symptoms” of psychotic disorders, are particularly relevant. Interestingly, human studies also reveal that men with psychosis show higher levels of negative symptoms than women [8], in further agreement with our observation of sex-dependent effects of adolescent THC on social behavior. Altogether, these findings highlight pronounced sex-dependent effects of adolescent THC on locomotor, social, and cognitive behaviors at adulthood.

In adults, reduced phosphorylation of mitochondrial Complex I (CI) subunits has emerged as a key target of THC-induced mitochondrial dysfunction, leading to altered behaviors [5]. CI consists of multiple conserved subunits forming an L-shaped structure with a peripheral arm (N and Q modules) for NADH oxidation and ubiquinone reduction, and a membrane-embedded P module for proton pumping [9]. Given its central role, CI dysfunction represents the most common mitochondrial-linked disorder in humans [10]. We recently showed that THC-induced instability of astrocytic CI, mediated by dephosphorylation of its NDUFS4 subunit, underlies THC-induced social deficits in adult animals [5]. Therefore, we asked whether adolescent THC exposure might similarly affect NDUFS4 phosphorylation, thereby determining the long-term behavioral consequences of cannabinoid intoxication. By quantifying total and phosphorylated NDUFS4 protein in hippocampus and prefrontal cortex at two time points, we firstly found interesting sex- and age-dependent differences between adolescent and adult stages (Fig. 1H-I), which warrant further investigation in future studies. However, our purpose was to investigate the impact of adolescent THC administration (1 and 5 mg/kg): immediately after adolescent exposure (PND46) and at early adulthood (PND70). In male mice, adolescent THC 5 mg/kg significantly reduced NDUFS4-Ser173 phosphorylation in hippocampus (Fig. 1J) and prefrontal cortex (Fig. 1K) but not in the cerebellum (Fig. 1L) at PND46. In contrast, THC 1 mg/kg also reduced NDUFS4-Ser173 phosphorylation in prefrontal cortex (Fig. 1K) but increased it in the hippocampus (Fig. 1J) without any effect in the cerebellum (Fig. 1L).Thus, we decided to continue with the use of THC 5 mg/kg to evaluate if these changes were maintained at adulthood or present in female mice. Notably, THC-induced reduction of NDUFS4-Ser173 phosphorylation was not present neither at adult stages (Fig. 1M) nor in female mice both at adolescent and adult stages (Fig. 1N). Altogether, these results point to a sex-dependent and transient mitochondrial signature of adolescent THC exposure.

To test the causal role of astroglial NDUFS4-Ser173 phosphorylation in the long-term behavioral impact of adolescent THC exposure, we intravenously (retro-orbital) delivered a systemically-acting adeno-associated viral vector (AAV-PHP.eB) to drive astrocyte-specific expression of a phosphomimetic NDUFS4 mutant (AAV-GFAP-NDUFS4-PM) in PND25 male mice (Fig. 1O-P). Following repeated adolescent THC exposure, locomotion and social interaction were assessed at adulthood (Fig. 1O). NDUFS4-PM overexpression did not affect social interaction (AAV-Control (AAV-Control-Vehicle and AAV-NDUFS4-Vehicle):0,71 ± 0,03; AAV-NDUFS4-PM:0,75 ± 0,04) and locomotion (AAV-Control (AAV-Control-Vehicle and AAV-NDUFS4-Vehicle):135 ± 22; AAV-NDUFS4-PM:146 ± 11), but it fully prevented long-term THC-induced deficits in sociability and locomotor activity (Fig. 1Q-R). These findings causally implicate astroglial NDUFS4 phosphorylation as a key mediator of adolescent THC’s long-term behavioral impact.

## Discussion

Our results provide a causal mechanistic bridge between adolescent cannabis intoxication and the long-term neurobehavioral consequences previously shown by epidemiological associations and preclinical studies. By identifying a transient, developmentally timed mitochondrial vulnerability in astrocytes, our work underscores how brief disruptions of bioenergetic signaling can durably alter circuit maturation [11]. Mechanistically, we propose a mitochondria-astrocyte-neuron pathway in which THC-triggered reduction in astroglial NDUFS4 phosphorylation destabilizes Complex I, reduces ROS production [12], and disrupts astrocytic metabolic support, ultimately impairing the proper development of neuronal circuits underlying sociability and other behavioral functions. However, our results do not allow us to definitively establish whether astrocytes are the sole or primary cellular effectors of THC-induced behavioral alterations. Female resilience may stem from greater intrinsic antioxidant capacity and estrogen’s protective effects [13], explaining the observed sex specificity. Indeed, our data demonstrating that female mice are resistant to the THC-induced dephosphorylation of NDUFS4 align with previous reports indicating that females have greater mitochondrial capacity than males [14]. However, future experiments should better investigate the sex differences observed in our study and to directly link this potential greater capacity with the resilience to cannabis effects.

Altogether, our findings indicate that persistent effects of repeated adolescent THC exposure arise from a transient reduction in astroglial NDUFS4 phosphorylation, establishing a long-awaited mechanistic explanation of this phenomenon. Although future works should better explore the cellular and/or circuit changes linking a transient mitochondrial alteration during adolescence into adult behavioral deficits and to evaluate these effects at latter time points, this discovery might represent a first therapeutic entry point for the prevention and treatment of the long-lasting effects of adolescent cannabis use.

## Data Availability

The datasets generated and/or analyzed during the current study are available from the corresponding author upon request.
